# The locatability of Pearson algorithm for multi-source location in complex networks

**DOI:** 10.1038/s41598-023-32832-w

**Published:** 2023-04-07

**Authors:** Hong-Jue Wang, Zhao-Long Hu, Li Tao, Shuyu Shao, Shi-Zhe Wang

**Affiliations:** 1 School of Information, Beijing Wuzi University, Beijing, 101149 People’s Republic of China; 2 College of Mathematics and Computer Science, Zhejiang Normal University, Jinhua, 321004 People’s Republic of China; 3 School of Economics, Liaoning University, Shenyang, 110000 People’s Republic of China

**Keywords:** Complex networks, Nonlinear phenomena

## Abstract

We study locating propagation sources in complex networks. We proposed an multi-source location algorithm for different propagation dynamics by using sparse observations. Without knowing the propagation dynamics and any dynamic parameters, we can calculate node centrality based on the character that positive correlation between inform time of nodes and geodesic distance between nodes and sources. The algorithm is robust and have high location accuracy for any number of sources. We study locatability of the proposed source location algorithm and present a corresponding strategy to select observer nodes based on greedy algorithm. All simulations on both model and real-world networks proved the feasibility and validity of this algorithm.

## Introduction

In our modern society, propagation dynamics taking place on complex networks are ubiquitous^1,2^. Examples include cascading effects diffusing in power grids^3^, epidemic disease^4^ or rumor^5^ propagating among contact relationships, computer virus^6^ or malware spreading through the Internet^3,7^. Locating propagation source efficiently and accurately has a wide range of applications. In epidemiology, researchers can obtain significant information about the disease by locating the source of epidemics. We can find out who start a rumor by identifying the rumor source in online social networks^8^, and prevent many baleful effects by locating the sources of online computer virus.

To date, a large number of approaches have been proposed to identify single propagation source in complex networks and these algorithms can usually obtain high location accuracy by traversing all nodes in network^9–13^. While in reality, many propagation dynamics often initiated from multiple sources^3,5,14^, if we traverse all nodes of network, the time complexity of the algorithm will increases exponentially with the increase of the number of sources. So multi-source locating problem has a greater challenge than single source locating. So far, little attention has been paid to multiple source location problem. In reference^14^, a multi-source location algorithm based on Jordan center has been proposed, while the number of sources was needed in advance. Some algorithms have been presented based on belief propagation^15,16^, these algorithms have satisfactory performance on the tree structure networks. A clustering method was proposed in^17^, while the algorithm had high complexity by traversing all combination of nodes in complex networks. Besides, Wen-Xu Wang has proposed two algorithms based on backward spreading^6^ and controllability theory^18^ successively, while these methods are limited to diffusion dynamics and need to know all parameters of propagation delays.

An important problem for source location using partial observer nodes is locatability of algorithms^19^. Locatability refers to whether the source centrality indexes of all nodes are different for a given set of observer nodes. The purpose of the locatability problem is to find the minimum set of observer nodes which can satisfy the source locatability condition and improve the source location accuracy as much as possible. In general, if we have access to information about all nodes in complex network, then the source can be identified naturally, because the message first appear at the source. However, for large-scale complex networks, obtaining complete propagation information is often costly or impossible. Therefore, how to obtain the least number of observer nodes to satisfy source locatability condition and obtain high-precision source location results is an important scientific problem in the direction of source location in complex networks. In fact, the selection of observer nodes has already attracted wide attention in the early stage. Literature^12^ use observer nodes to locate source, and the observer nodes were selected randomly according to the degree of nodes. Based on literature^12^, literature^20^ proposed an observer node selection strategy based on coverage rate. It was found in literature^21^ that the sum of distance difference between source and observer nodes increased, and the location accuracy of the source increased. Literature^22^ also proposed a method to select observer nodes based on the maximum and minimum values of diffusion delay variance. Based on the observability theory of complex networks, literature^18^ provide a selection method of the least observer nodes. Literature^23^ focuses on the relationship between network diffusion delay noise and observer nodes selection, and propose observer nodes selection strategies for low and strong noise diffusion respectively. In recent years, the selection of observer nodes has attracted more and more attention^24,25^. Literature^24^ proposes an observer nodes selection strategy that uses greedy algorithm to maximize diffusion delay variance. In literature^26^, the author found that there was an important correlation between source location accuracy and observer nodes selection, and proposed an optimal observer node selection strategy based on naive Bayes model. In addition, in literature^19,27^, the selection of observer nodes was discussed respectively according to the characteristics of the source location algorithms, and the locatability index closely related to the source location algorithm and network structure was defined. Greedy algorithm was used to maximize the locatability index, so as to obtain the optimal combination of observer nodes. Although the research on locatability problem started earlier, most of them use the network structure to compute node centrality for selecting observer nodes, and ignoring the influence of characteristics of source location algorithm.

To sum up, although source location of complex networks has made great progress, there are still many difficulties, the most important of which are the following three aspects: (1) Most source location algorithms are proposed for specific propagation models, and the propagation parameters of models in the network need to be known before execute source location algorithm. In fact, it is difficult to fully understand the properties and parameters of different propagation models, which will bring great challenges to data acquisition and source location algorithms. It is not wise to establish a source location algorithm for each propagation models. We need more location algorithms applicable to different propagation models. This allows locating sources without knowing the parameters of the propagation model. (2) Most of the source location algorithms only consider the single source situation. Although many of the single source location algorithms have been successfully extended to the multi-source situation, most of them need to know the number of sources and need to cooperate with the community detection technology for multi-source location. In fact, it is generally difficult to get the number of sources before all source location is completed, and community location is a complex and difficult work, which may eventually increase the cost and difficulty of source location and reduce the efficiency. (3) Most observer nodes selection methods only consider the factors of network structure, ignoring the influence of the characteristics of source location algorithm on the distribution of observer nodes and the source location accuracy. When selecting observer nodes, some source location algorithms only consider the influence of network structure on the accuracy of source location, and determine the merits of observer nodes simply by using the properties of node degree, node betweenness, node clustering coefficient, etc., ignoring the requirements of the algorithm on observer nodes.

On the problem of most multiple source location methods mentioned above, we proposed an algorithm for different propagation dynamics include Diffusion^28^ and SI^2^. In reference^2^, Dirk Brockmann and Dirk Helbing proposed a source location algorithm based on the positive correlation of epidemic arrival time and effective distances which on a global perspective of demographics and mobility between different communities. Inspired by this work, in communities, the time of nodes receiving message from source are certainly proportional to the geodesic distance between nodes and source, so we use Pearson correlation coefficient of the time and distance to determine all sources of complex network. We use area under the receiver operating characteristic curve (AUC) to quantify the accuracy of our algorithm. In addition, we discuss the locatability of source locating algorithms and propose an strategy to select advisable observer nodes based on a sufficient and necessary condition for the locatability of Person algorithm and the defined measurement index of observer nodes.

The rest of this paper is arranged as follows. First, two propagation models including Diffusion and SI dynamics are introduced. Second, we define the new Pearson multi-source locating algorithm and then discuss the locatability of algorithms and propose a strategy to select advisable observer nodes. Finally, we conclude this paper.

## Propagation model

Our goal is to locate the source of propagating message which taking place on an undirected complex network using only limited observed knowledge. The topological structure of the complex network $G=(V,E)$,with $n=|V|$ nodes and $m=|E|$ edges is assumed to be known, where *V* is the set of nodes and *E* is the set of edges.The connection of the network *G* can be represented as an adjacency matrix *A*, and its element ${a_{i,j}}$ is 1 when a link between nodes $v_i$ and $v_j$ exists and 0 otherwise. If $a_{i,j}=1$, nodes $v_i$ and $v_j$ are neighbors to each other. The degree ${k_i}$ is the number of neighbors of node $v_i$ .

### Diffusion dynamics

Epidemic spreading, rumor propagation and financial crises cascading can be modeled as diffusion-like dynamics^28^. During diffusion dynamics, there are two possible states for any node: informed (the node has received the message from sources) or uninformed (the node has not yet received the message from sources). We suppose message propagating through the shortest paths in complex network. At first, source $s \in S$ begin to spread the message at initial time $t_s$, which also called its informed time, where *S* is the set all sources. After an arbitrary node $v_i$ received message for the first time at $t_{v_i}$, it will transfer the message to all its neighbors. And the uninformed neighbor $v_j$ of node $v_i$ receive the message at time ${t_{{v_i}}}+\theta$, where $\theta$ is the propagation time delay of the edge between $v_i$ and $v_j$.

### SI dynamics

SI dynamics is a type of epidemic model, which is employed to describe the infection processes of nodes in networks^9,29^. In SI dynamics, nodes are initially uninformed and can receive message from neighbours along with the propagation of message. Once a node receive message, it remains informed forever. The state of an arbitrary node $v_i$ is denoted as $C_i$, where

$$\begin{aligned} {C_i} = \left\{ {\begin{array}{*{20}{c}}{0,}&{}{uninformed}\\ {1,}&{}{informed}\end{array}} \right. \end{aligned}$$

the probability of an arbitrary node $v_i$ being informed by its neighbors at time *t* is

$$\begin{aligned} p_i^{0 \rightarrow 1}(t) = 1 - {(1 - {\lambda _i})^{\sum \limits _{j = 1,j \ne i}^N {{a_{ij}}{C_j}(t)} }} \end{aligned}$$

where ${\lambda _i}$ is the informed rate of $v_i$, ${C_i}(t)$ stands for the state of node $v_i$ at time *t*, and the superscript $0\rightarrow 1$ denotes the change from uninformed state (indicated as 0) to informed state (indicated as 1).

**Figure 1:**
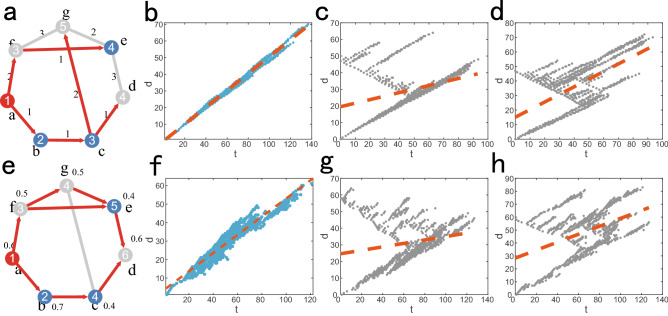
Property of propagation models. (**a**) is an example of diffusion dynamics in a simple network. Red nodes are sources of message and links with arrows represent the direction of message propagation. Numbers in the center of nodes are their informed time. Blue nodes are observer nodes and we will use their informed time to locate source nodes. Numbers beside links are their propagation time delay. During diffusion dynamics, message is transmitted along the shortest path. Source node *a* begin to spread message at initial time ${t_a}=1$, nodes *b*, *c*, *d*, *e*, *f* and *g* receive message from their neighbour nodes at informed time ${t_b}=2$, ${t_c}=3$, ${t_d}=4$, ${t_e}=4$, ${t_f}=3$, ${t_g}=5$ respectively. (**b**)–(**d**) are positive correlation between inform time of nodes and geodesic distance between nodes and source. We execute experiment on ER random network with $n = 1000$ and the average degree is $\left\langle k\right\rangle = 2$. The sources are selected randomly. Abscissa is inform time of nodes and ordinate is geodesic distance between nodes and sources. The orange line is the fitting result. (**b**) is the result of diffusion dynamics with one source, the time delay of edges follows Gaussian distribution $N(u,{\sigma ^2})$ with $u = 2$ and $\sigma = 0.25$. (**c**) and (**d**) are the results with two sources at once, (**c**) corresponds to the first source and (**d**) corresponds to the second one. (e) is an example of the SI dynamics. Numbers beside nodes are informed rate. Source node *a* begin to spread message at initial time ${t_a}=1$, nodes *b*, *c*, *f*, *g* are infected by their neighboring nodes with their respective informed rate. Node *e* is infected by nodes *f* and *g* with probability 0.64, node *d* is infected by nodes *e* and *c* with probability 0.84. (**f**)–(**h**) are positive correlation between inform time of nodes and geodesic distance between nodes and source of SI dynamics on ER random network. We set informed rate as $\lambda = 0.5$ for SI dynamics.

Figure 1 is schematic diagram of diffusion and SI dynamics. (a) and (e) are details of diffusion and SI dynamics respectively. From (b) and (f), we can see that the time of nodes receiving message from source are proportional to the geodesic distance between nodes and source, so reference^30^ use this characteristic to locate single source, while we can see from (c), (d), (g) and (h) for multi-source location problem, the positive correlation property significantly reduce and is invalid.

**Figure 2:**
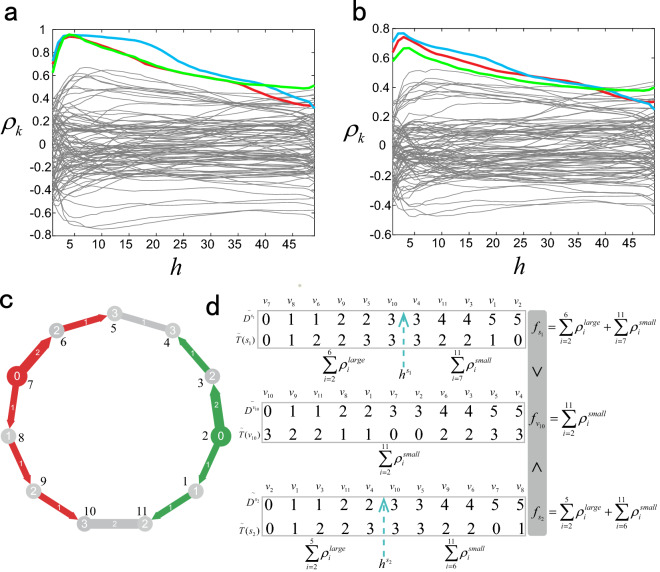
Description of multi-source location method. (**a**)–(**b**) are the relationship between Pearson centrality ${\rho _k}$ and threshold *h*, we simulate diffusion and SI dynamics on ER random network with $n = 100$ and the average degree is $\left\langle k\right\rangle = 4$. (**a**) is diffusion dynamics with $u = 2$ and $\sigma = 0.25$ and (**b**) is SI with informed rate as $\lambda = 0.5$. Three sources and fifty percent observer nodes are selected randomly. Abscissa is threshold *h* of ${\widetilde{\pmb {D}}^{k}_h}$ and ${\widetilde{\pmb {T}}}_h$, ordinate is pearson centrality ${\rho _k}$. Different curves correspond to different nodes, three curves with red, blue and green corresponding to three source nodes. (**c**) is an example of network. Numbers in the center of nodes are their informed time and numbers beside the nodes are their labels. The red node (node $v_7$) is source $s_1$ and the green node (node $v_2$) is source $s_2$, their initial time are both 0. Red and green links with arrows represent the direction of message propagation from source $s_1$ and $s_2$ respectively. (**d**) is description of calculating index *f*. We consider the *f* value of source $s_1$, node $v_{10}$ and source $s_2$ of network in (**a**). We sort ${{\pmb {D}}^{s_1}}$, ${{\pmb {D}}^{v_{10}}}$ and ${{\pmb {D}}^{s_2}}$ in ascending order as ${\widetilde{\pmb {D}}^{s_1}}$, ${\widetilde{\pmb {D}}^{v_{10}}}$ and ${\widetilde{\pmb {D}}^{s_2}}$ respectively. Then we get the corresponding vectors of nodes informed time as ${\widetilde{\pmb {T}}}({s_1})$, ${\widetilde{\pmb {T}}}({v_{10}})$ and ${\widetilde{\pmb {T}}}({s_2})$. Firstly, for source $s_1$, we set a threshold $h \in (2,11)$ and calculate Pearson centrality of the vectors ${\widetilde{\pmb {D}}^{s_1}_h}$ and ${\widetilde{\pmb {T}}}_h(s_1)$ as ${\rho _h}$, where ${\widetilde{\pmb {D}}^{s_1}_h}$ is comprised of the first *h* elements in ${\widetilde{\pmb {D}}^{s_1}}$, and we can get ${\widetilde{\pmb {T}}}_h(s_1)$ in the same way. When threshold *h* is less than ${h^{s_1}}$, because elements in ${\widetilde{\pmb {D}}^{s_1}_h}$ and ${\widetilde{\pmb {T}}}_h(s_1)$ have positive correlation, we can get large Pearson centrality value ${\rho _h^{large}}$. And when *h* is larger than ${h^{s_1}}$, because some nodes received message from other sources and their corresponding elements will reduce the positive correlation of ${\widetilde{\pmb {D}}^{s_1}_h}$ and ${\widetilde{\pmb {T}}}_h(s_1)$, then we will get small Pearson centrality value ${\rho _h^{small}}$. We can do the same operation on node $v_{10}$ and source $s_2$ as above. Because ${\widetilde{\pmb {D}}^{v_{10}}_h}$ and ${\widetilde{\pmb {T}}}_h(v_{10})$ have no distinct positive correlation, we can only get small Pearson centrality value for all *h* of node $v_{10}$. So we use the sum value *f* of all ${\rho _h}$ to measure the likelihood that a node is a source. And the *f* of sources are larger than which of non-source nodes, e.g., $f_{s_1}>f_{v_{10}}$ and $f_{s_2}>f_{v_{10}}$.

## Multi-source location algorithm

We set ${{\pmb {D}}^k} = {\left[ {d_1^k,d_2^k, \ldots ,d_{n - 1}^k,d_n^k} \right] ^{\textrm{T}}}$ as the vector of geodesic distance between node ${v_k}$ and all other nodes, where $d_i^k$ is the distance between nodes ${v_i}$ and ${v_k}$. We sort ${{\pmb {D}}^k}$ in ascending order and get ${\widetilde{\pmb {D}}^{k}}={\left[ {d_{{i_1}}^k,d_{{i_2}}^k, \ldots ,d_{{i_{n - 1}}}^k,d_{{i_n}}^k} \right] ^{\textrm{T}}}$, where $d_{{i_j}}^k \le d_{{i_{j + 1}}}^k$. So the informed time vector of nodes is ${\widetilde{\pmb {T}}}= {\left[ {{t_{{i_1}}},{t_{{i_2}}},\ldots ,{t_{{i_{n - 1}}}},{t_{{i_n}}}} \right] ^{\textrm{T}}}$, where ${t_{{i_j}}}$ represents the informed time of node ${v_{{i_j}}}$. From a statistical point of view, the time of nodes receiving message from source are proportional to the geodesic distance between nodes and source, see Fig. 1b and f. If there is only one source in network, and ${v_k}$ is the source, then ${\widetilde{\pmb {D}}^{k}}$ and ${\widetilde{\pmb {T}}}$ have a coincident monotonic increasing. So the Pearson correlation coefficient of ${\widetilde{\pmb {D}}^{k}}$ and ${\widetilde{\pmb {T}}}$ is used as the source indicator, which called Pearson centrality and marked as ${\rho _k}$. The Pearson centrality ${\rho _k}$ will be high when ${\widetilde{\pmb {D}}^{k}}$ and ${\widetilde{\pmb {T}}}$ have a similar rank, and low otherwise. In this paper, the closer that ${\rho _k}$ to 1, the more likely ${v_k}$ be the source. For source node *s*, its Pearson centrality is larger than which of non-source nodes. It is noteworthy that the Pearson centrality can be calculated without knowing the kind of propagation dynamics.

**Figure 3:**
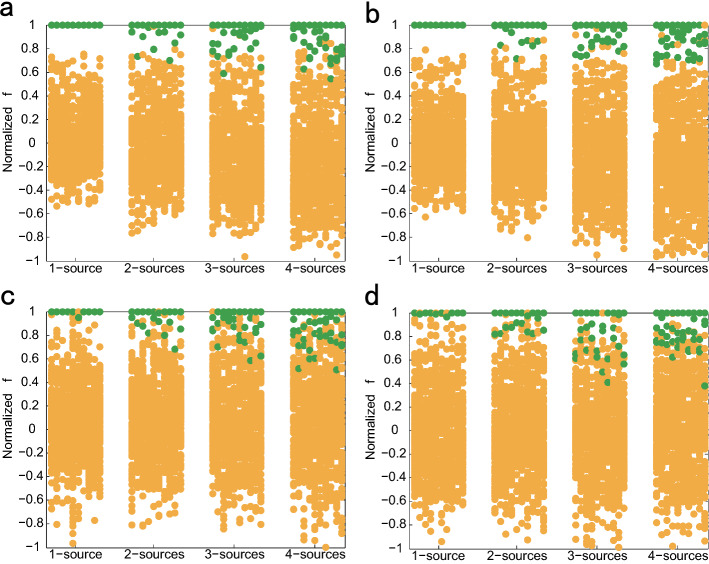
Normalized *f* value of nodes. We execute experiment on ER and BA networks with $n = 100$ and average degree $\left\langle k \right\rangle = 4$. For diffusion dynamics in (**a**) and (**b**), the time delay of edges follows Gaussian distribution $N(u,{\sigma ^2})$ with $u = 2$ and $\sigma = 0.25$ and we set informed rate as $\lambda = 0.5$ for SI dynamics in (**c**) and (**d**). Randomly select $50\%$ of nodes as observer nodes. Abscissa is the number of sources, ordinate is normalized *f* value. We randomly selected sources have the same initial time as 0 and 10 repetitive experiments are executed for each number of source. Clearly, for all cases, the values *f* of true sources remain larger than which of non-sources. All *f* values are normalized and each value is divided by the maximum *f* value of each node. (**a**) and (**c**) are BA network, (**b**) and (**d**) are ER network.

For multiple sources, only based on the value of ${\rho _k}$, it is hard for us to determine which node are the source directly. Because some corresponding nodes of the elements which contained in vector ${\widetilde{\pmb {T}}}$ of a source received message from other sources, that is to say the elements in ${\widetilde{\pmb {T}}}$ are not monotonically increasing and then ${\widetilde{\pmb {T}}}$ are inconsistent with the monotonicity of ${\widetilde{\pmb {D}}^{k}}$, see Fig. 1c,d,g,h. Therefore, the Pearson centrality of sources will decrease. Faced with this problem, we set a threshold $h \in (2,n)$ and calculate Pearson centrality $\rho _k^h$ of the vectors ${\widetilde{\pmb {D}}^{k}_h}$ and ${\widetilde{\pmb {T}}}_h$, where ${\widetilde{\pmb {D}}^{k}_h}= {\left[ {d_{{i_1}}^k,d_{{i_2}}^k, \ldots ,d_{{i_{h - 1}}}^k,d_{{i_h}}^k} \right] ^\mathrm{{{T}}}}$ and ${\widetilde{\pmb {T}}_h}= {\left[ {{t_{{i_1}}},{t_{{i_2}}}, \ldots ,{t_{{i_{h - 1}}}},{t_{{i_h}}}} \right] ^\mathrm{{{T}}}}$. As a matter of fact, for a source *s*, it has a perfect threshold ${h^s}$, and the corresponding nodes of the elements less than ${h^s}$ received message from *s*, the corresponding nodes of the elements larger than ${h^s}$ received message from other sources. So the Pearson centrality value of $h \le {h^s}$ is large and the value of $h > {h^s}$ is small. In fact, the threshold ${h^s}$ can be regarded as the radius of a source infection range. In order to highlight centrality values of sources, we accumulate the large Pearson centrality values of $h \le {h^s}$ by summing all values of different threshold *h*:

1 $$\begin{aligned} {f_k} = \sum \limits _{h = 1}^n {\rho _k^h} \end{aligned}$$

Here the value of ${f_k}$ is the sum of value $\rho _k^h$ of different threshold *h*. So, the node ${v_k}$ with large ${f_k}$ value is more likely to be a source. The detailed description of calculating *f* is shown in Fig. 2. (a) and (b) in Fig. 2 are the simulation results. It can be seen that the Pearson centrality $\rho _k$ of the source nodes first increases and then decreases with the increase of the threshold *h*. When threshold *h* reaches the maximum, it is equivalent to directly calculating the Pearson centrality $\rho _k$ of the informed time vector and the vector of geodesic distance between nodes and source. (c) and (d) are the schematic diagram of calculating index ${f_k}$ proposed in this paper. Figure 3 shows the normalized *f* value of all nodes for different propagation dynamics on ER model network. Clearly, for most cases, the centrality values of sources are larger than that of non-sources, this enabling us to correctly identify all sources. Algorithm 1 is detailed step of multi-source location algorithm.
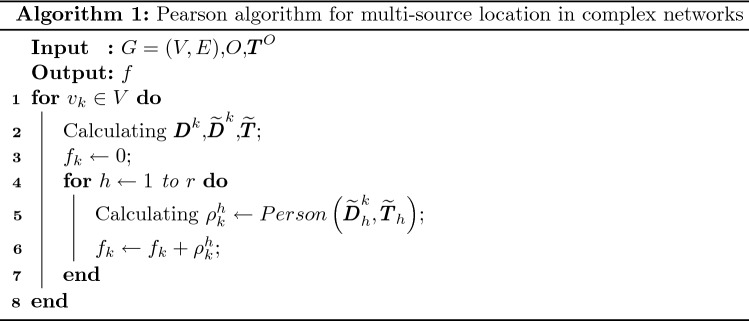


## Results

During diffusion or SI dynamics, we monitor observer nodes $O = \{ {o_1},{o_2}, \ldots ,{o_r}\}$ and record their informed time ${{\pmb {T}}^O} = \left[ {{t_{{o_1}}},{t_{{o_2}}}, \ldots ,{t_{{o_r}}}} \right]$. After all nodes of *V* have been informed, the observed knowledge $(O,{{\pmb {T}}^O})$ is used to calculate *f* values of all nodes. In order to quantify the validity and efficiency of the proposed multi-source location algorithm based on limited observer nodes, we study the success rate of locating sources on both model and real networks for different propagation dynamics. Model networks are BA scale-free network (BA)^6^ and ER random network (ER) with $n = 100$ and average degree $\left\langle k \right\rangle = 4$. Real static networks include social contact networks, biological networks and transport networks. Detailed information of all the networks is shown in Table 1. During simulations in this paper, observer nodes and source are selected randomly. Here a standard metric, area under the receiver operating characteristic curve (AUC)^29^ is used to quantify the accuracy of our algorithm. We first rank the nodes based on their *f* values in ascending order and result in a new candidate list. *AUC* value is calculated by two indexes, true positive rate (TPR) and false positive rate (FPR).

2 $$\begin{aligned} TPR(l) = \frac{{TP(l)}}{{{n_s}}} \end{aligned}$$

where *TP*(*l*) is the number of true sources in the top *l* nodes of candidate list, ${n_s} = \left| S \right|$ is the size of set *S*.

3 $$\begin{aligned} FPR(l) = \frac{{FP(l)}}{{n - {n_s}}} \end{aligned}$$

where *FP*(*l*) is the number of false positives in the top *l* nodes of candidate list. The abscissa of receiver operating characteristic curve is *FPR* and ordinate is *TPR*, *AUC* is the area under this curve. The higher value of *AUC*, the better location performance of algorithm. All results *AUC* are mean value of 200 repetitive simulations.

**Figure 4:**
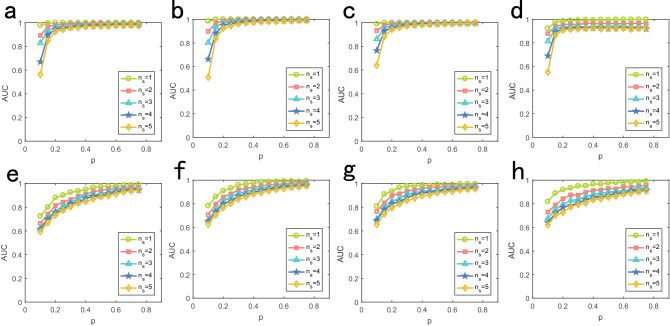
Performance of the multiple source location with different number of sources on model and real networks. We set the number of sources from 1 to 5 and different numbers corresponding to different color. All initial time of sources are set as 0. Abscissa is fraction of observer noddes and ordinate is AUC value. (**a**)–(**d**) Diffusion dynamics on BA, ER, Football and Santa Fe respectively. (**e**)–(**h**) are SI dynamics.

**Figure 5:**
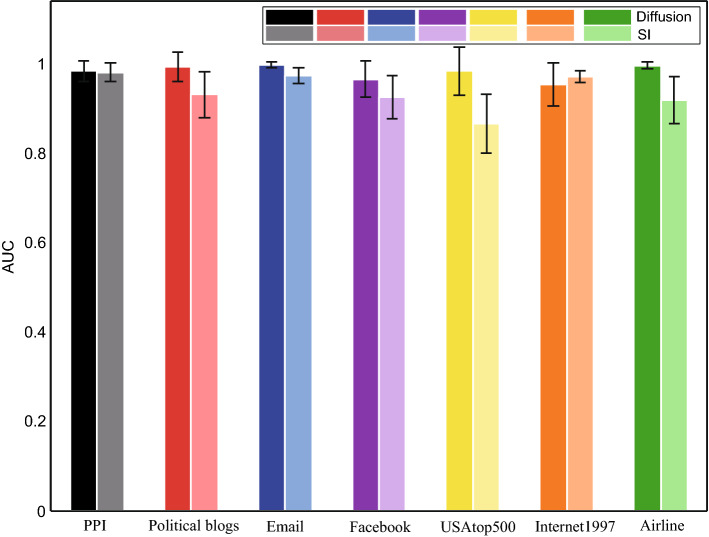
Performance of sources location on seven additional real static networks. For diffusion dynamics, the time delay of edges follows Gaussian distribution $N(u,{\sigma ^2})$ with $u = 2$ and $\sigma = 0.25$ and we set informed rate as $\lambda = 0.5$ for SI dynamics. Randomly select $30\%$ of nodes as observer nodes. We set two sources and which are randomly selected. All values are average of 100 simulations, and the error bars are standard deviations.

For multi-source location problem, Fig. 4 shows the accuracies of source location on model and real networks respectively. We set $u = 2$ and $\sigma = 0.25$ for the time delay distribution of diffusion dynamics, and set identical $\lambda =0.5$ for SI dynamics. All sources have the same initial time. As we can see, the performance of our method increase with the fraction of observer nodes, and the increase of number of sources enhances the challenge of successfully locating the sources. For diffusion dynamics, we can obtain $AUC > 0.8$ by using only $20\%$ observer nodes. For SI dynamics, we can obtain $AUC > 0.6$ by using only $10\%$ observer nodes. On model networks, the sources of BA network is more challenging to locate than ER network. On real networks, the performance of the algorithm on Football is better than that of Santafe. In addition, we also simulated our algorithm on seven other large-scale networks, see Fig. 5. Clearly, we can get satisfactory performance on all networks by only observing 30% nodes.

**Figure 6:**
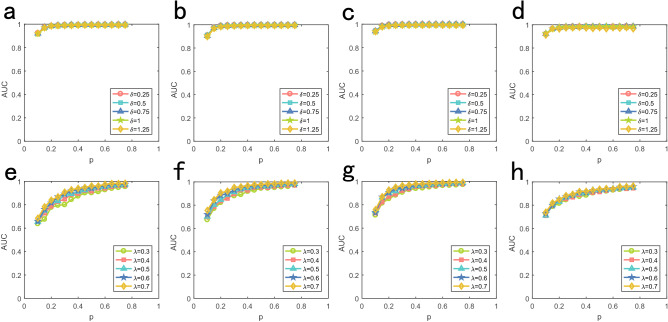
Performance of the multiple source location with different model propagation parameters. Abscissa is fraction of observer nodes and ordinate is AUC value. All initial time of sources are set as 0. (**a**)–(**d**) are diffusion dynamics on BA, ER, Football and Santa Fe respectively. Curves of different colors corresponding to different standard deviation $\sigma$ with the same $u = 2$ of diffusion process. (**e**)–(**h**) are SI dynamics. Curves of different colors corresponding to different informed rate $\lambda$ of SI epidemic model.

The accuracies of some algorithms are sensitive to noisy propagation dynamics and have low robustness^6^. We study the robustness of our algorithm by simulating on model and real networks for different propagation parameters. We set the number of sources as 2 and all sources have identical initial time as 0. For the time delay distribution of diffusion dynamics, we set identical $u = 2$ and different $\sigma = 0.25,0.5,0.75,1,1.5$. As is shown in Fig. 6, the performance of our method is not visibly affected by the parameter $\sigma$. For SI dynamics, we set different informed rate as $\lambda = 0.3,0.4,0.5,0.6,0.7$. As is shown, the performance of our method decrease with the decrease of $\lambda$. Because low informed rate lead to more disordered informed time of nodes. Clearly, for all different $\sigma$ of diffusion dynamics, only approximately $10\%$ observer nodes can receive relatively high accuracy $AUC > 0.8$, and $AUC > 0.6$ for different $\lambda$ of SI dynamics. To sum up, the performance of our algorithm indicate relative high robustness.

**Figure 7:**
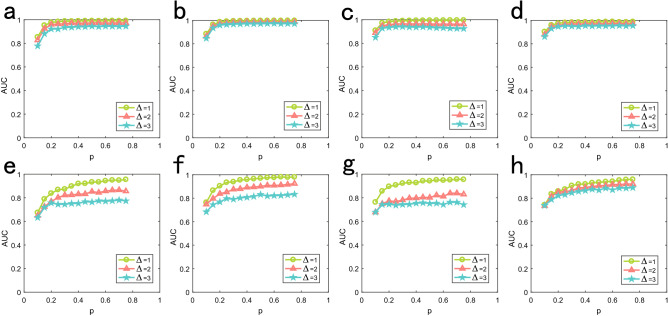
Performance of sources location with different initial time. We set the number of sources as 2, curves of different colors corresponding to different initial time difference between two sources. (**a**)–(**d**) are Diffusion dynamics on BA, ER, Football and Santa Fe respectively. (**e**)–(**h**) are SI dynamics.

Multi-source location problem of diffusion process is a great challenge, especially for the situation of different initial time. For two sources situation, we set the initial time as ${{\pmb {T}}^s} = \left[ {{t_{{s_1}}},{t_{{s_1}}} + \Delta \cdot u} \right]$ for diffusion dynamics and ${{\pmb {T}}^s} = \left[ {{t_{{s_1}}},{t_{{s_1}}} + \Delta } \right]$ for SI dynamics, where $\Delta = 1,2,3$. We set $u = 2$ and $\sigma =0.25$ for the time delay distribution of diffusion dynamics and $\lambda = 0.5$ for SI dynamics. Figure 7 shows the performance of sources location with different initial time on model and real networks. Clearly, the greater $\Delta$ of two sources, the more difficult for us to locate the source successfully.

**Figure 8:**
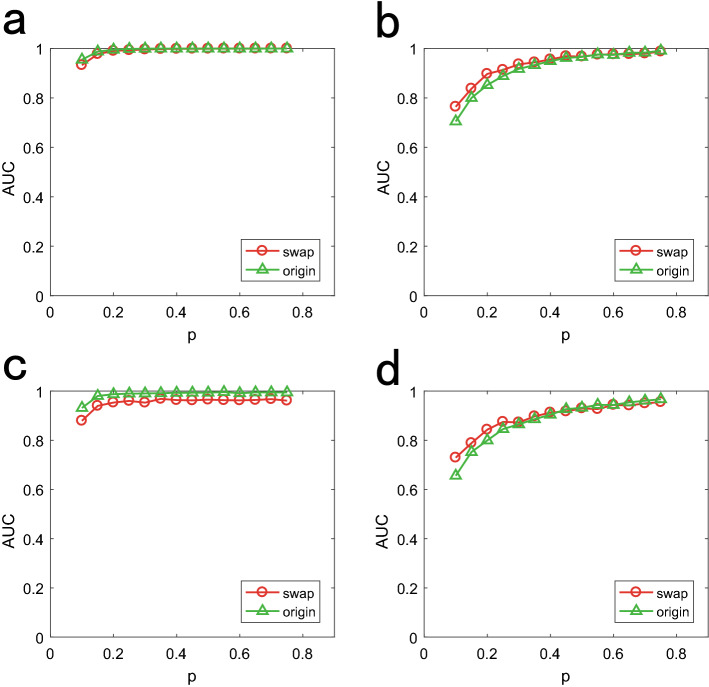
Performance of our algorithm on original network (green) and fully randomly swaped version of it (red). We set the number of sources as 2. (**a**), (**b**) are diffusion and SI dynamics on Football nework respectively. (**c**), (**d**) are diffusion and SI dynamics on Santafe nework respectively.

In addition, we also study how other network properties except average degree affect our method accuracy. We randomly swap all edges and preserve the degree sequence of real networks. The results are summarized in Fig. 8. We set $u = 2$ and $\sigma =0.25$ for the time delay distribution of diffusion dynamics, and set identical $\lambda = 0.5$ for SI dynamics. We consider two sources situation. As we can see, for all networks and propagation dynamics, there is no marked difference between the two cases, so the network topology properties except average degree do not play a significant role in our method performance.

**Table 1:** Description of real networks.

Networks		*n*	*m*	$\left\langle k\right\rangle$	Description
Football^31^		115	613	10.6609	The network of American football games, Fall 2000
Santa Fe^31^		118	200	3.3729	Scientific collaboration network of the Santa Fe. Institute
Airline^32^		332	2126	12.8072	US air transportation network
USAtop500^33^		500	2890	11.920	US air transportation network by considering the 500 airports with largest traffic
Email^34^		1133	5451	9.6222	Email communication network
PPI^35^		2375	11693	9.8467	ProteinCprotein interaction network
Facebook^36^		4039	88,234	43.6910	The online social network as similar as Face-book
Internet1997^37^		3015	5156	3.4202	Autonomous Systems topology of the Internet
Political blogs^38^		1224	19,090	27.3552	Hyper links between web logs on US politics

## Locatability

Locatability is an important problem, which will directly affect the observer node selection strategy of the algorithm^19,27^. Using the source locatability of an algorithm, we can choose the appropriate observer nodes, which can save resources and improve the source location accuracy. Different algorithms may have different source locatability condition, so different algorithms have different observer node selection strategies. The locatability of the algorithm in this paper will be discussed in detail next. We are talking about source locatability in the ideal state, that is, the network edge delay is the same. Therefore, the locatability of source node is completely determined by network structure and source location algorithm.

### Locatability condition

For two nodes *u* and *v*, ${\pmb {d}}_o^v$ and $d_o^u$ are the distance vectors of the two nodes respectively, and the element $d_{{o_i}}^v$ in vector ${\pmb {d}}_o^v$ is the shortest path between node *v* and observer node ${o_i}$. For Person centrality, that is, ${\rho ^p}\left( v \right) = person({{\pmb {T}}^O},{\pmb {d}}_o^v)$, where *person* is Pearson correlation coefficient. Here we also give a sufficient and necessary condition for the locatability of Person algorithm:

4 $$\begin{aligned} d_{{o_i}}^v \ne k \cdot d_{{o_i}}^u + c \end{aligned}$$

where *k* is any positive real number and *c* is any real number. Apparently, $d_{{o_i}}^v = k \cdot d_{{o_i}}^u + c \Leftrightarrow person({{\pmb {T}}^O},{\pmb {d}}_o^u) = person({{\pmb {T}}^O},k \cdot {\pmb {d}}_o^u + c) = person({{\pmb {T}}^O},{\pmb {d}}_o^v) \Leftrightarrow {\rho ^P}\left( v \right) = {\rho ^P}\left( u \right)$. In other words, if $d_{{o_i}}^v = k \cdot d_{{o_i}}^u + c$, then we have $person(d_{{o_i}}^v,d_{{o_i}}^u)=1$, that is, nodes *u* and *v* are indistinguishable. In order to be able to distinguish all the nodes by Pearson centrality, we use the number of node pairs that satisfy $person(d_{{o_i}}^v,d_{{o_i}}^u) \ne 1$ as the locatability index of Pearson algorithm which denoted as *x*.

**Figure 9:**
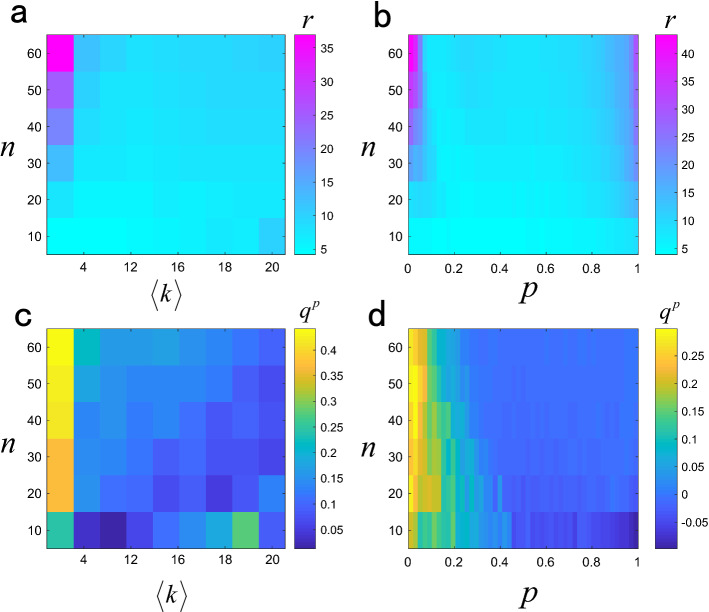
Relationship between locatability and network parameters. (**a**), (**b**) are relationship between the number of locatability observer nodes and network parameters on BA and ER networks respectively. The abscissa is the average degree of the network in (**a**) and the adding link probability in (b), ordinate is network size *n*, and the colors in heat map are the number of locatability observer nodes. (**c**), (**d**) are relationship between measurement index $q^p$ of locatability observer nodes and network parameters. The colors in heat map are the measurement index $q^p$. (**c**) is BA and (**d**) is ER network.

### Selecting observer nodes

According to the above analysis, it is reasonable to believe that each algorithm should have its own relatively feasible observer node selection strategy, and the strategy are corresponding to the sufficient condition of source locatability. In fact, although the source location algorithm can ensure that the centrality of source nodes are greater than that of the non-source nodes in most cases, there are still cases where the centrality of source nodes and non-source nodes are the same, which is not conducive to clearly distinguish the source nodes from non-source nodes. Therefore, it is necessary to use the observer nodes based on locationability sufficient and necessary condition to eliminate the case of the same centralities. So the calculated centralities of the source nodes based on locationability sufficient and necessary condition are completely greater than that of non-source nodes.Then the source location accuracy of the observer nodes based on locationability sufficient and necessary condition should be higher than that of the randomly selected observer node. So as long as we find the minimum set of observer nodes satisfying the sufficient condition for locationability, from a theoretical point of view, the centrality of source nodes obtained by using these observer nodes should be greater than that of non-source nodes. It is worth pointing out that, because the selection of observer nodes is also related to network structure, propagation model parameters and other factors, the observer nodes we choose can only improve the location accuracy to a certain extent, but may not be the optimal combination among all observer node combinations. Because the combination of observer nodes that makes all nodes in the network distinguishable is not unique, obtaining such a set of observer nodes is a combinatorial optimization problem and is NP hard. Therefore, we propose an observer nodes selection strategy based on locationability sufficient condition and locatability index *x* by using greedy algorithm. Before giving the observer nodes selection strategy, we first give an indicator to measure the quality of observer nodes. Suppose that the set of observer nodes set is *O*, then the shortest paths of all observer nodes and all nodes in the network form a shortest path matrix

5 $$\begin{aligned} D = \left[ {\begin{array}{*{20}{c}} {d_{{o_1}}^{{v_1}}}&{}\quad {d_{{o_1}}^{{v_2}}}&{}\quad \cdots &{}\quad {d_{{o_1}}^{{v_n}}}\\ {d_{{o_2}}^{{v_1}}}&{}\quad {d_{{o_2}}^{{v_2}}}&{}\quad \cdots &{}\quad {d_{{o_2}}^{{v_n}}}\\ \vdots &{}\quad \vdots &{}\quad \vdots &{}\quad \vdots \\ {d_{{o_k}}^{{v_1}}}&{}\quad {d_{{o_k}}^{{v_2}}}&{}\quad \cdots &{}\quad {d_{{o_k}}^{{v_n}}} \end{array}} \right] \end{aligned}$$

*D* is the matrix of $k \times n$, and *k* is the number of observer nodes in the network. ${{\pmb {d}}_i}$ is the $i-th$ column of *D*. Then the measurement index of the observer nodes of the Person algorithm is

6 $$\begin{aligned} {{q^p} =1- \frac{2}{{n(n - 1)}}\sum \limits _{i < j} {Person\left( {{{\pmb {d}}_i},{{\pmb {d}}_j}} \right) }} \end{aligned}$$

The smaller ${q^p}$, the better combination of observer nodes, and the greater the difference between ${{\pmb {d}}_i}$, and the calculated Person centrality of the source nodes will be much different from that of the non-source nodes, which can better highlight the source nodes. Then the observer nodes selection strategy is described as Algorithm 2.

**Figure 10:**
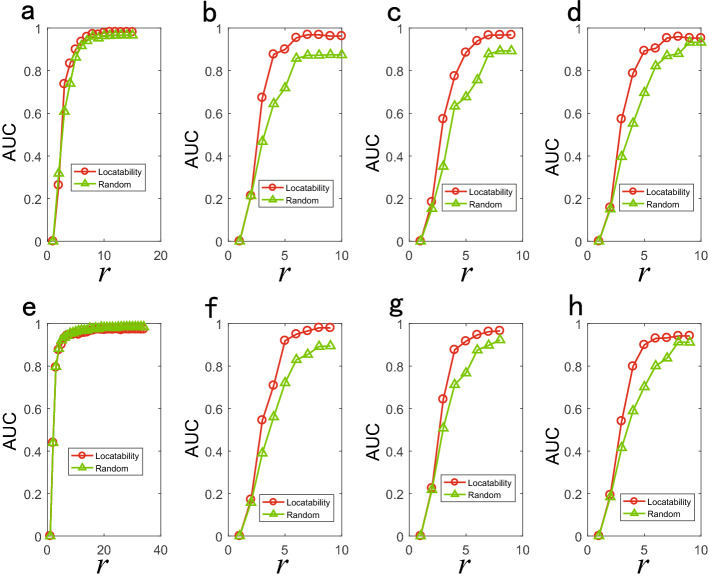
Locating source results of locatability and random strategy of diffusion dynamics on model networks. Abscissa is the number of observer nodes r and ordinate is AUC value. We simulate diffusion epidemic on model network with 50 nodes. We set $u = 2$ and $\sigma =0.25$ for the time delay distribution and two sources. (**a**)–(**d**) are the results of BA networks and correspond to network average degree $\left\langle k\right\rangle =2, 4, 6$ and 8 respectively. (**e**)–(**h**) are the results of ER networks and correspond to adding link probabilities 0.05, 0.25, 0.45 and 0.65 respectively.

**Figure 11:**
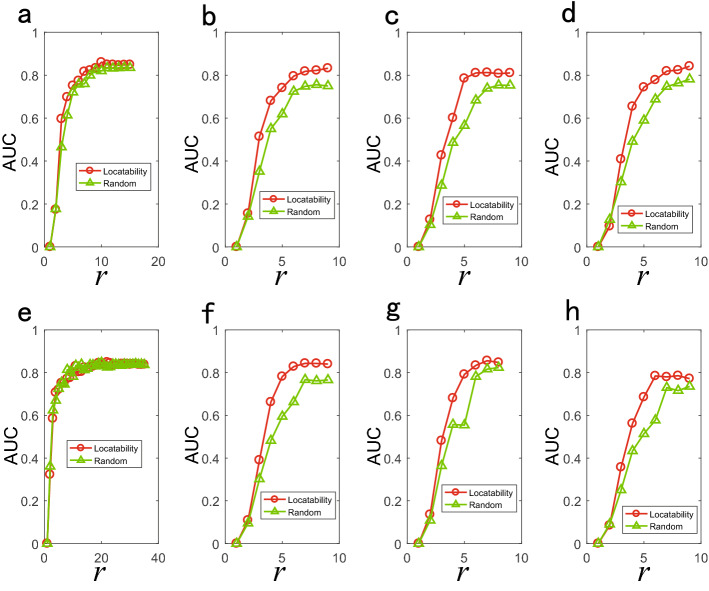
Locating source results of locatability and random strategy of SI dynamics on model networks. Abscissa is the number of observer nodes *r* and ordinate is AUC value. We simulate SI epidemic on model network with 50 nodes and set $\lambda = 0.5$ and two sources. (**a**)–(**d**) are the results of BA networks and correspond to network average degree $\left\langle k\right\rangle =2, 4, 6$ and 8 respectively. (**e**)–(**h**) are the results of ER networks and correspond to adding link probabilities 0.05, 0.25, 0.45 and 0.65 respectively.

We discuss the relationship between parameters of model networks and the number of locatability observer nodes. It can be seen form Fig. 9 that the number of locatability observer nodes in BA network increases with the increase of network scale and decreases with the increase of network average degree $\left\langle k \right\rangle$. The measurement index ${q^p}$ corresponding to the locatability observer nodes increases with the increase of network scale, and decreases with the increase of network average degree. The number of locatability observer nodes in ER network increases with the increase of network scale, and decreases first and then increases with the increase of adding link probabilities *p*. The index ${q^p}$ corresponding to the locatability observer nodes increases with the increase of network scale, and decreases with the increase of adding link probabilities. We compare the source location results of locatability observer nodes with the results of randomly selected observer nodes. Figures 10 and 11 show the source location results of diffusion and SI dynamics respectively, and it is found that the source location accuracy can be improved by locatability observer nodes.
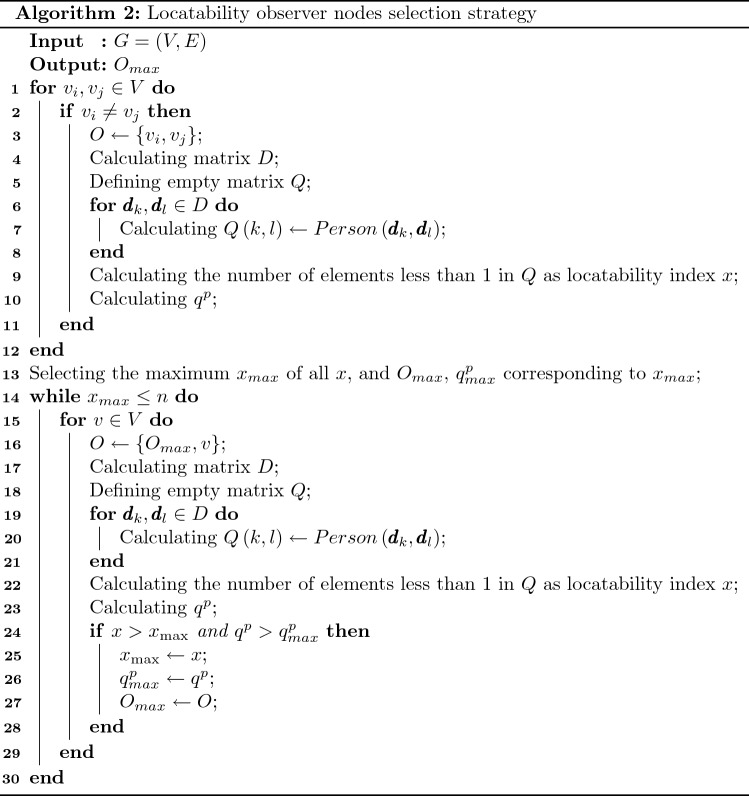

## Discussions

In this work, we investigated multi-source location problem in complex networks and proposed a sources location method based on the positive correlation between inform time of nodes and geodesic distance between nodes and source. The proposed method is appropriate for different propagation dynamics. Without knowing what kind of propagation dynamics, the proposed method can locate sources by using limited observed information of observer nodes. We study the locating accuracy of our algorithm on different model and real networks under different propagation dynamics including Diffusion and SI. We study the influence of different number of sources, different parameters of propagation dynamics (the robustness of the algorithm), different initial time and different properties of networks on locating accuracy. We also discuss the locatability of source locating algorithms and propose selecting observer nodes locatability strategy based on a sufficient and necessary condition for the locatability of Person algorithm and the defined measurement index of observer nodes. The observer nodes selected by locatability strategy can effectively improve the accuracy of locating source. We simulated on model and empirical networks respectively and finally verified that the proposed source locating algorithms and selecting observer nodes strategy are feasible and effective. Regardless of this, the proposed method still have some room for improvement. First, the observer placement strategy need to study in depth, because it is still hard for us to select the minimum number of observer nodes in an arbitrary network for preferable locating accuracy. Second, in this paper, we only study the performance of our algorithm on two familiar propagation dynamics, we hope our algorithm can be used to more propagation dynamics. Third, we need more efforts to reduce the time complexity.

## Data Availability

The datasets used and analysed during the current study available from the corresponding author on reasonable request.
